# Knockdown of POLQ interferes the development and progression of hepatocellular carcinoma through regulating cell proliferation, apoptosis and migration

**DOI:** 10.1186/s12935-021-02178-2

**Published:** 2021-09-13

**Authors:** Qi Pan, Lu Wang, Yu Liu, Min Li, Yao Zhang, Wei Peng, Tan Deng, Mei-Ling Peng, Jin-Qiong Jiang, Jiao Tang, Jingjing Wang, Hua-Xin Duan, Sha-Sha Fan

**Affiliations:** 1 Oncology Department, The First Affiliated Hospital of Hunan Normal University, Hunan Provincial People’s Hospital, Key Laboratory of Study and Discovery of Small Targeted Molecules of Hunan Province, Hunan Normal University, Changsha, 410000 Hunan China; 2 Department of Pathology, The First Affiliated Hospital of Hunan Normal University, Hunan Provincial People’s Hospital, Changsha, 410000 Hunan China; 3 Department of Hepatic Surgery, Department of Oncology, Fudan University Shanghai Cancer Center, Shanghai Medical College, Fudan University, Shanghai, 200032 China; 4 Department of Oncology, The Second Xiangya Hospital of Central South University, Changsha, 410000 Hunan China

**Keywords:** Hepatocellular carcinoma, POLQ, Cell proliferation, Cell apoptosis, Cell migration

## Abstract

**Background:**

DNA Polymerase Theta (POLQ) is a DNA polymerase involved in error-prone translesion DNA synthesis (TLS) and error-prone repair of DNA double-strand breaks (DSBs), whose function in hepatocellular carcinoma has not been investigated.

**Methods:**

In the present study, both the data collected from the Cancer Genome Atlas (TCGA) and our group’s results showed higher POLQ expression in HCC tissues than the para-cancerous tissues, which was associated with higher malignancy and poor prognosis. POLQ knockdown HCC cell model (shPOLQ) was constructed along with the corresponding negative control (shCtrl) through lentivirus infection for loss-of-function study.

**Results:**

We found that, upon knockdown of POLQ, the proliferation and migration of HCC cells decreased and apoptosis percentage increased. Moreover, the percentage of cells in G2 phase significantly increased in shPOLQ group compared with shCtrl group. Xenografts in mice grafted with shPOLQ cells grew much slower than that transplanted with shCtrl cells, and expressed lower Ki67 level. Furthermore, an apoptosis-related signaling array was used to explore the involvement of downstream signaling pathways, suggesting the enhanced phosphorylation of HSP27 and JNK, and the de-activation of mTOR, PRAS40, ERK1/2 and STAT3 pathways.

**Conclusions:**

Collectively, our study revealed that POLQ may participate in the development of HCC, depletion of which may be a promising treatment strategy for HCC.

## Introduction

In the past decades, malignant tumors have become one of the leading causes of human death worldwide. According to the latest statistics, among all malignant tumors, the incidence and mortality of liver cancer ranks sixth and fourth, respectively [1, 2]. There are approximately 840,000 newly discovered liver cancer cases and 780,000 liver cancer related deaths each year, imposing a heavy burden on society and public health [2]. Among all types of primary liver cancer, hepatocellular carcinoma (HCC) accounts for more than 80%, which is the most important histological type [3]. Clinically, the treatment of HCC is a combination of surgical resection and chemotherapy [4]. Although surgical resection has a good curative effect on early HCC, due to the small diameter of tumor and the lack of typical clinical symptoms and signs, the patient's tumor frequently has progressed to the advanced stage at the time of diagnosis. Recently, the application of molecular targeted drugs such as Sorafenib and Lenvatinib paved new path for the treatment of HCC [5]. However, the improvement of HCC patients’ prognosis is still far from satisfactory [6]. Therefore, getting deeper insight into the underlying mechanism in HCC development could be benefit for facilitating more effective therapy against HCC and improving patient prognosis and quality of life.

DNA polymerase theta (POLQ) is an evolutionarily conserved protein encoded by the *POLQ* gene in mammalian genomes. POLQ is the defining enzyme for a pathway of DNA double-strand break (DSB) repair termed "alternative end-joining" (altEJ) or "theta-mediated end-joining" [7]. The enzyme has several unique properties, including low fidelity and the ability to insert and extend past abasic sites and thymine glycollesions. It is important for maintaining genetic stability of cells and protecting cells from DNA breaks formed by ionizing radiation and topoisomerase inhibitors, breaks arising at stalled DNA replication forks, breaks introduced during diversification steps of the mammalian immune system, and DSB induced by CRISPR-Cas9 [8]. Three "insertion" sequence elements present in POLQ cannot be found in any other A-family DNA polymerase, and it has been proposed that they may lend some unique properties to POLQ [9]. Studies concerning the biological functions of POLQ in human diseases is rarely seen. High POLQ expression was directly associated with defective DNA replication fork progression and chromosomal damage [10]. The upregulation of POLQ in breast cancer was deduced to play a great role in increasing intrinsic radio-sensitivity [11, 12]. Despite, to the best of our knowledge, the relationship between POLQ and HCC has not been well documented.

In this study, the role of POLQ in HCC was initially investigated by bioinformatics based on The Cancer Genome Atlas (TCGA) database and immunohistochemical staining of HCC tissues and normal tissues. Loss-of-function studies were carried out both in vitro and in vivo to explore the regulatory effects of POLQ on HCC development and progression. The underlying mechanism was explored through the application of an apoptosis-related signaling pathways antibody array analysis.

## Materials and methods

### Cell culture and cell infection

Hepatocellular carcinoma cell lines Huh7, MHCC-97L, SK-HEP-1, BEL-7404 and HepG2 were purchased from BeNa Technology (Hangzhou, China) and cultured in a cell incubator with a humid condition at 37 °C with 5% CO_2_. Cells were cultured in 90% RPMI-1640 (31800022, GIBCOA) supplemented with 10% fetal bovine serum (FBS) (10099-141, GIBCO). The shPOLQ SK-HEP-1 and BEL-7404 cells and the control cells were established by knockdown lentiviral vector (1 × 10^8^ TU/mL) infection with ENI.S and Polybrene. After culturing for 72 h, the infection efficiency was detected by GFP fluorescence.

### Clinical specimens

To assess the protein POLQ (NM_199420) expression level, we analyzed 180 pathological sections via IHC assay. Endogenous peroxidase was deactivated by 3% H_2_O_2_ and non-specific-binding sites were blocked with 4% skim milk powder for 30 min. Sections were immersed into antigen-retrieval solution for antigen retrieval at 95 °C for 30 min. The sections were then incubated with primary antibody for POLQ protein overnight at 4 °C. The corresponding secondary antibody was then added to incubate for 30 min at room temperature. All slides were stained DAB solution and counterstained with 10% Harris hematoxylin. IHC results were judged by positive cell score and staining color intensity score. POLQ expression positive cell scored as follows: negative: no positive signal; positive: 1 point, 0% < positive cells accounted for < 25%; 2 points, 25% ≤ positive cells proportion < 50%; 3 points, 50% ≤ positive cells < 75%; 4 points, positive cells proportion ≥ 75%. The intensity was scored as: 0 point, no positive signal; 1 point, weak; 2 points, moderate; 3 points intense.

### Lentiviral vector construction

According to the principle of RNA interference sequence design, multiple 19–21 nt were designed based on the POLQ template RNA interferes with the target sequence. After the evaluation and determination of the design software, the following sequences are selected as interference targets. Human-POLQ-1, AAGGATAAAGCTTAATACAGA, Human-POLQ-2, TATTTCAAACTTGGAGACAAA, Human-POLQ-3, GTGGAAGAAGCCAGAATGATT. POLQ cDNA was generated by PCR, and subsequently cloned into BR-V108. The plasmids were extracted with TIANgel Midi Purification Kit (TIANGEN, DP209-03) and EndoFree Maxi Plasmid Kit (TIANGEN, DP117) and POLQ lentivirus was packaged.

### qRT PCR

Total RNA was extracted from cell following Sigma Trizol instructions, the concentration and quality of RNA was determined by Nanodrop 2000/2000c spectrophotometer (Thermo Fisher Scientific). RNA was reverse transcribed into cDNA by M-MLV kit (Promega). Real-time PCR was performed with SYBR premix EX Tap (TAKARA). PCR cycling program was conducted, holding at 95 °C for 30 s, two steps PCR at 95 °C for 5 s, 60 °C for 30 s, dissociation at 95 °C for 15 s, 60 °C for 30 s, and 95 °C for 15 s. The relative quantitative analysis in gene expression data was analyzed by 2^−ΔΔCt^ method and the melt curve was drawn. The primers sequences were showed as follow and GAPDH was served as inner control:

POLQ Forward primer: 5’-TCCTACACCCATTCCAACATCTG-3’,

Reverse primer: 5’-GTCTTTGAACCCATTTCTACTCCC-3’;

GAPDH Forward primer: 5’-TGACTTCAACAGCGACACCCA-3’,

Reverse primer: 5’-CACCCTGTTGCTGTAGCCAAA-3’.

### Western blot assay

Total protein from shPOLQ and shCtrl SK-HEP-1 and BEL-7404 cells were subjected to western blot analysis. Briefly, after bathed in boiling water for 10 min, the total cellular proteins were subjected to SDS-PAGE (10%). After transferring to polyvinylidene difluoride (PVDF) membranes, blots were incubated with 5% BSA (Gibco) in Tris-buffered saline containing 0.5% Tween 20 for 60 min and incubated overnight at 4 °C on a rocker with the following primary antibodies: anti-POLQ (biorbyt, # orb48495), anti-mTOR (abcam, # ab2732), anti-p-mTOR (abcam, # ab137133), anti-CCND1 (CST, # 2978), anti-Bcl-2 (abcam, # ab196495), anti-c-Myc (CST, # 5605) and anti-GAPDH (Bioworld, # AP0063). Following washing three times with TBST for 5 min, the plots were then incubated with horseradish peroxidase (HRP) conjugated goat anti-rabbit IgG polyclonal secondary antibody (1:3000, Beyotime, # A0216) at room temperature for 1 h. Amersham’s ECL + plusTM western blotting system kit was used for color developing. Signals were detected with enhanced chemiluminescence, using GAPDH as the internal standard (Kodak), and analyzed by ImageJ software.

### Cell apoptosis detection

Apoptotic cells were identified using Annexin V-FITC Apoptosis kit (eBioscience, 88–8007-74) by flow cytometric methods. Transfected cells and corresponding control cells were plated in a six-well plate (2 mL/well) and cultured to 85% confluence. Cells were collected and centrifuged at 1300 rmp for 5 min, supernatant was discarded and cells were washed with 4 °C pre-cooled D-Hanks (pH = 7.2–7.4) and resuspended in the 1 × binding buffer. 10 μL Annexin V-APC was stained for 15 min in the dark. Apoptotic cells were measured using Guava easyCyte HT FACScan (Millipore) to assess the apoptotic rate.

### Colony formation assay

Transfected cells and corresponding control cells suspension was seeded into 6-well plates with 500 cell/well. Continuously cultured for 14 days, cells were immobilized with 4% paraformaldehyde and stained with GIEMSA (DingGuo Biotechnology). Colonies (containing more than 50 cells) were photographed with a digital camera. The proliferative potential was assessed by counting clone numbers.

### MTT assay

The viability of lentivirus infected cells was assessed using MTT assay. 0.1 mL cell suspension was seeded into a 96-well plate (2 000 cells/well) (Cornning, # 3599) and cultured. Before detection, 20 μL 5 mg/mL MTT (3-(4,5-dimethylthiazol-2-yl)-2,5-diphenyl tetrazolium bromide) (Genview, # JT343) solution was added to each well reacted for 4 h, and 100 μL DMSO were added to lyse the Formazan crystal. OD490 was determined for each well using a microplate reader (Tecan infinite) at the same time of 5 continuous days. The cell viability ratio was calculated by the following formula: cell viability (%) = OD (shPOLQ)/OD (shCtrl) × 100%.

### Wound healing assay

Infected cells and corresponding control cells (1 × 10^6^ cells) were seeded in 6-well plates and cultured for 24 h to 90% confluence. Then, wounds were created using a 200 µL plastic pipette tip, and images were acquired at 0, 8 h and 24 h after wounding using a microscope. The speed of wound closure in multiple fields reflected the migratory ability of the tumor cells.

### Transwell assay

Infected cells and corresponding control cells (1 × 10^4^) were seeded in the upper chamber of a Transwell filters (Corning) with polycarbonate membrane (8 µm). 600 µL medium with 30% FBS was added into the lower chamber and cultured at 37 °C for 24 h. Then the chamber was reversed on the absorbent paper to remove the medium and non-migrated cells were removed with cotton swabs. Migrated cells on the lower membrane surface were fixed and stained, 100 × and 200 × magnification pictures were plotted (five random fields per well were selected).

### Animal tumor formation experiment and vivo animal imaging

Sufficient number of cells were prepared, and the tumor cells in the logarithmic growth stage were digested with trypsin, and then the medium was completely suspended. Then, the cells was injected subcutaneously into the 4-week-old, female BALB/c nude mice (purchased from Shanghai Jiesijie Experimental Animal Co., Ltd.) and the tumor-forming condition was observed. The tumor size and mice weight were measured. Tumor size and animal weight were measured every other day. The animal experiments were approved by the Ethics Committee of Hunan Provincial People's Hospital. Before being executed, mice were intraperitoneal injected 0.7% pentobarbital sodium solution (10 μL/g, SIGMA). After completely entered the coma state, the mice were put into the darkroom of the IVIS Spectrum imaging instrument (Perkin Elmer) for live animals, in vivo imaging detection is performed on mice according to the established detection procedures to observe the fluorescence expression in mice.

### Statistical analyses

Each cell experiment was carried out at least 3 time under the same conditions. Qualitative data were shown as mean ± SD. The significance of the differences was determined using the two-tailed Student’s t test analyzed by SPSS version 21.0 with P values less than 0.05 as statistically significant. Mann–Whitney U and Pearson analysis were applied to explain the relationships between POLQ expression and tumor characteristics in patients with HCC.

## Results

### POLQ is upregulated in HCC and associated with HCC development

**Fig. 1:**
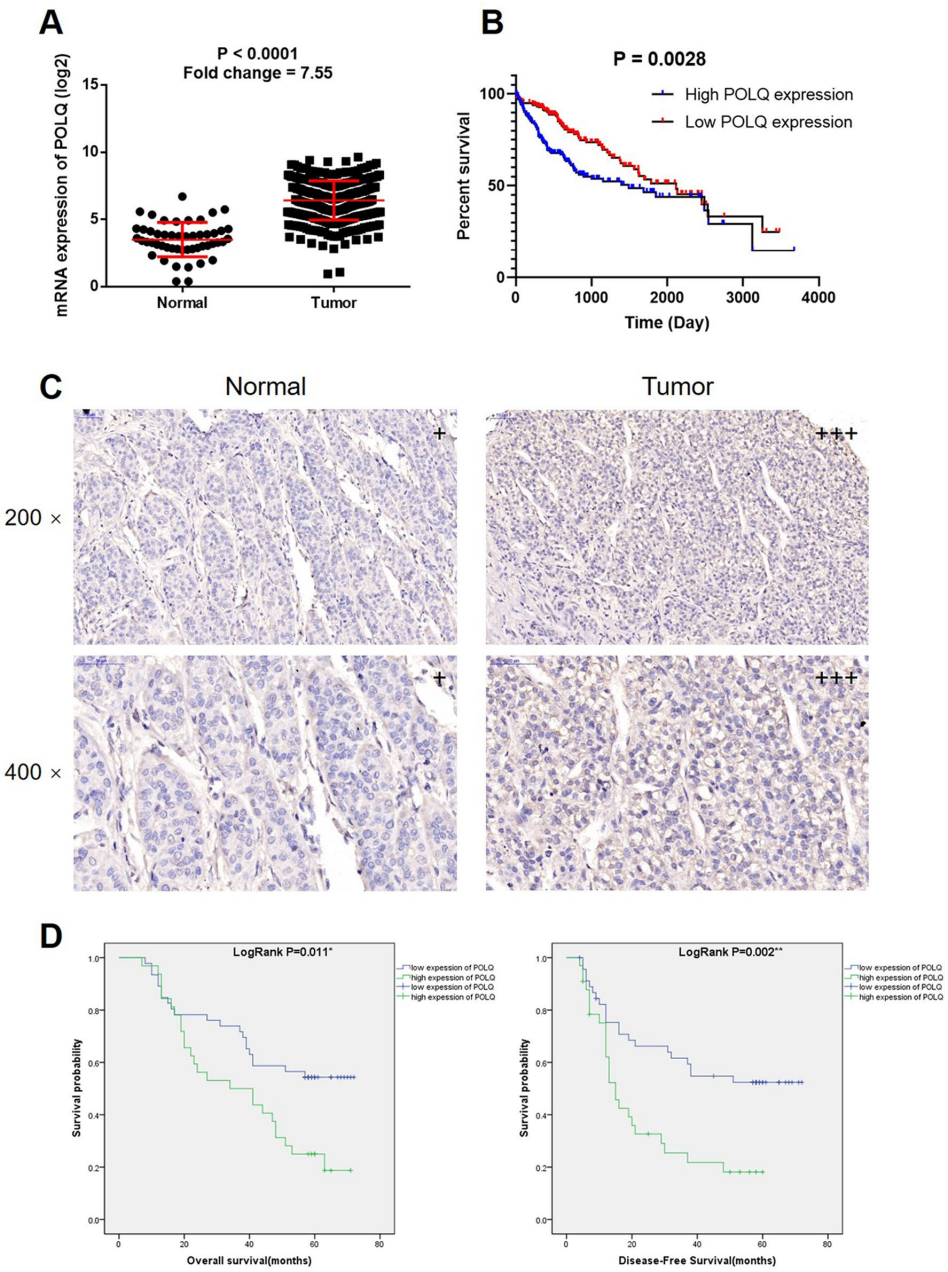
POLQ is upregulated in HCC and associated with HCC development. **A** The expression of POLQ in HCC tissues and normal tissues was collected from TCGA database and compared. **B** The correlation between POLQ expression and HCC patients’ prognosis was analyzed by Kaplan–Meier survival analysis based on data of TCGA database. **C** The expression of POLQ in HCC tissues and normal tissues was detected by IHC. **D** The relationship between POLQ expression and HCC patients’ survival was analyzed by Kaplan–Meier survival analysis with log rank test

First of all, the expression of POLQ in 50 normal tissues and 374 tumor tissues was collected from LIHC dataset of TCGA database. The results indicated the significantly upregulation of POLQ in HCC (Fig. 1A), which probably predicts the poor prognosis of HCC patients (Fig. 1B). Moreover, POLQ expression was further detected in 80 HCC tissues and 81 para-carcinoma tissues collected by our group. As shown in Fig. 1C and Table 1, POLQ was expressed in all the clinical specimens, and displayed relatively higher expression in HCC tissues, which was statistically significant. Further taking tumor characteristics into account, we demonstrated that high POLQ expression was significantly associated with more lesion number, higher risk of tumor recurrence, and higher expression of ALT and GGT (Tables 2 and 3). Finally, a survival analysis revealed the potential of POLQ as a prognostic indicator, high expression of which predicted poorer prognosis (Fig. 1D). Collectively, all the results suggested that POLQ may be involved in the development and progression of HCC.

**Table 1:** Expression patterns of POLQ in HCC tissues and normal tissues revealed in immunohistochemistry analysis

POLQ expression	Tumor tissue		Normal tissue	
	Cases	Percentage	Cases	Percentage
Low	47	58.8%	81	100%
High	33	41.2%	0	–

*P* < 0.001

**Table 2:** Relationship between POLQ expression and tumor characteristics in patients with HCC

Features	No. of patients	POLQ expression		*P* value
		low	high	
All patients	80	47	33	
Age (years)				0.114
<51	40	20	20	
≥51	40	27	13	
Gender				0.206
Male	65	36	29	
Female	15	11	4	
Cirrhotic nodule				0.198
No	11	7	4	
≤3 mm	33	22	11	
>3 mm	36	18	18	
Tumor number				0.001
≤1	65	44	21	
>1	15	3	12	
Grade				0.831
II	52	31	21	
III	28	16	12	
Stage				0.681
1	53	32	21	
2	27	15	12	
T Infiltrate				0.681
T1	53	32	21	
T2	27	15	12	
Tumor recurrence				<0.001
No	32	28	4	
Yes	48	19	29	
Liver cirrhosis				0.443
No	10	7	3	
Yes	70	40	30	
HBsAg				0.725
No	11	7	4	
Yes	69	40	29	
HBcAb				0.299
No	8	6	2	
Yes	70	39	31	
Total bilirubin				0.863
<17.1 μmol/L	59	35	24	
≥17.1 μmol/L	21	12	9	
ALT				0.013
<40 μ/l	35	26	9	
≥40 μ/l	45	21	24	
Serum albumin				0.546
<4 g/dl	22	12	10	
4 g/dl≤ALB<5.5 g/dl	57	34	23	
≥5.5 g/dl	1	1	0	
AFP				0.451
<20 μg/l	23	12	11	
≥20 μg/l	57	35	22	
GGT				0.025
<40 μ/l	23	18	5	
≥40 μ/l	57	29	28

**Table 3:** Relationship between POLQ expression and tumor characteristics in patients with HCC analyzed by Pearson correlation analysis

Tumor characteristics	index	
Tumor number	Pearson correlation	0.378
	Significance (two tailed)	0.001
	n	80
Tumor recurrence	Pearson correlation	0.477
	Significance (two tailed)	<0.001
	n	80
ALT	Pearson correlation	0.278
	Significance (two tailed)	0.012
	n	80
GGT	Pearson correlation	0.252
	Significance (two tailed)	0.024
	n	80

### The establishment of POLQ knockdown cell lines

**Fig. 2:**
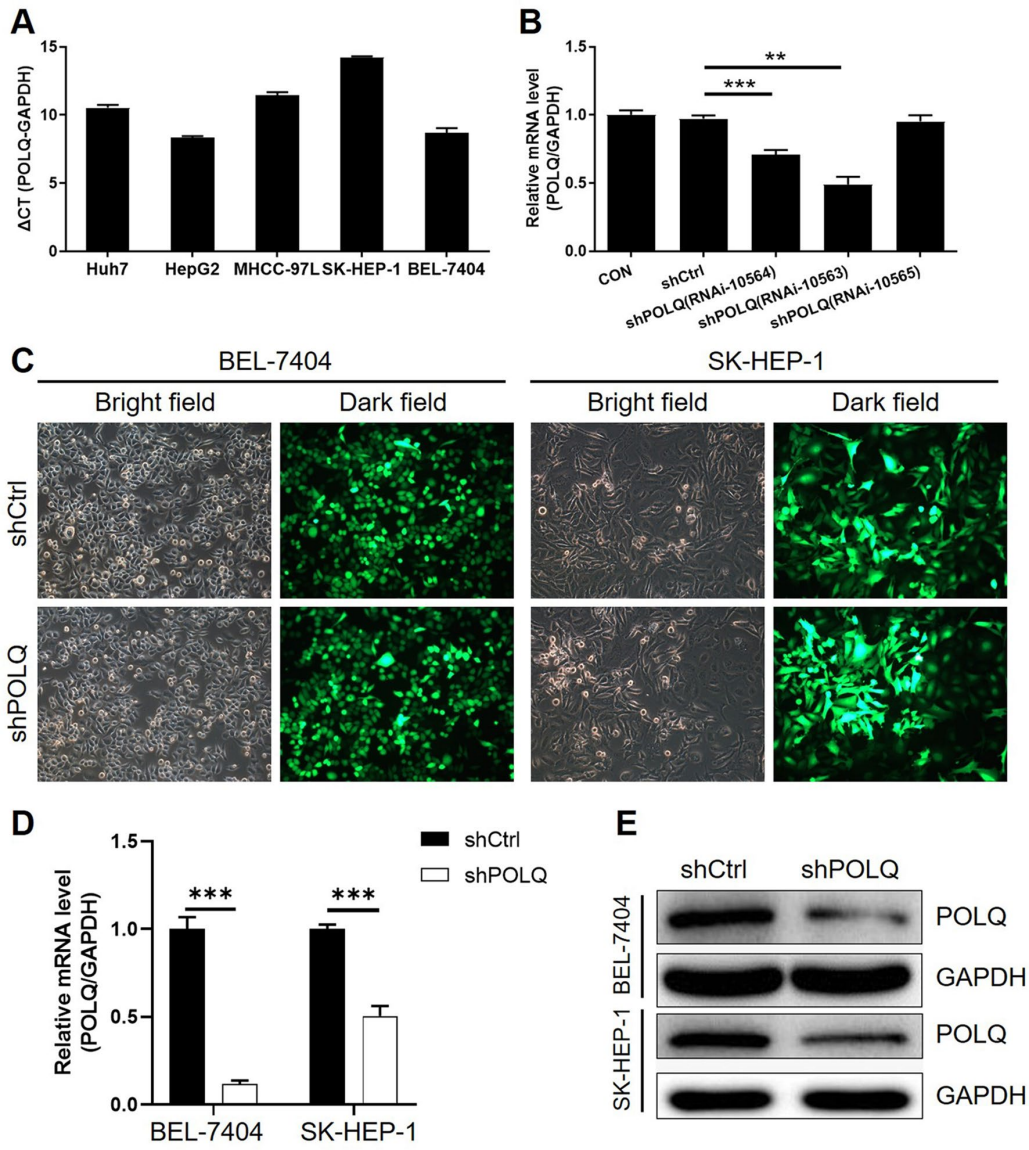
The establishment of POLQ knockdown cell lines. **A** The endogenous expression of POLQ in various HCC cell lines was detected by qPCR. **B** The knockdown efficiencies of shRNAs designed for silencing POLQ were evaluated by qPCR in SK-HEP-1 cells. **C** The transfection efficiencies of lentivirus plasmids in BEL-7404 and SK-HEP-1 cells were observed and measured through fluorescence imaging. **D**, **E** Knockdown of POLQ in HCC cells was verified by qPCR (**D**) and western blotting (**E**), respectively. Data = mean ± SD (n ≥ 3). ** P < 0.01, *** P < 0.001

Endogenous POLQ mRNA expression was tested in Huh7, HepG2, MHCC-92L, SK-HEP-1 and BEL-7404 cell lines (Fig. 2A), among which SK-HEP-1 and BEL-7404 cell lines were selected for constructing POLQ knockdown cell models. Figure 2B showed that, expression of POLQ in SK-HEP-1 cells were the best knocked down in shPOLQ (RNAi-10563) group with a knockdown efficiency of 49.6 (P < 0.01), therefore screening RNAi-10563 as the shRNA used in all the following experiments. The fluorescence of cells which were infected with shCtrl or shPOLQ plasmids for 72 h, observed by microscope demonstrated a > 80% efficiency of infection and the normal cell condition (Fig. 2C).Moreover, the mRNA and protein expression levels of POLQ were downregulated in BEL-7404 (Fig. 2D) and SK-HEP-1 (Fig. 2E), respectively, proving the successful construction of POLQ knockdown HCC cell lines.

### POLQ knockdown inhibited cell proliferation and induced cell apoptosis

**Fig. 3:**
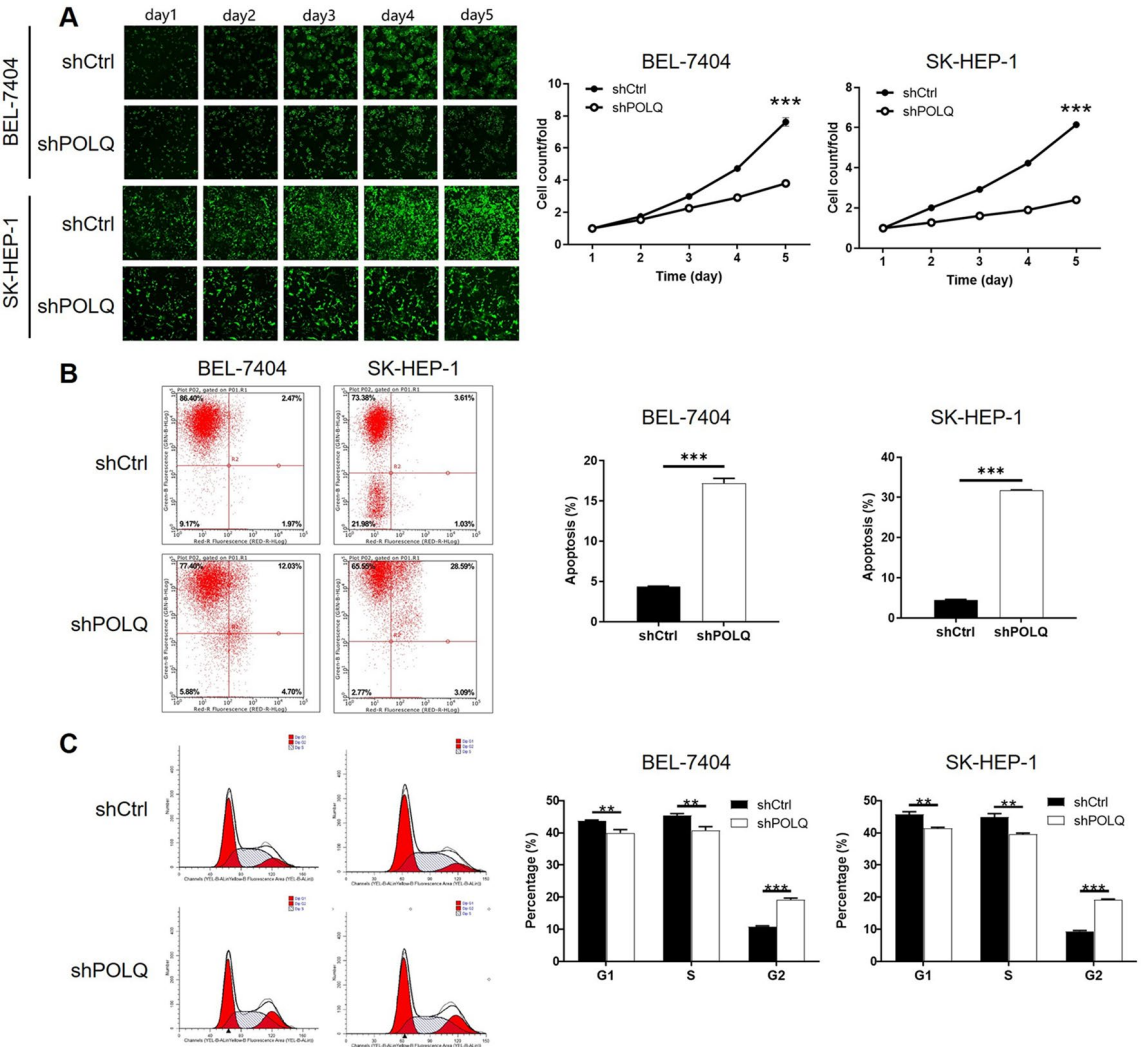
POLQ knockdown inhibited cell proliferation and induced cell apoptosis. **A** The inhibition of cell proliferation by POLQ knockdown in HCC cell lines was proved by Celigo cell counting assay. **B** The promotion of cell apoptosis by POLQ knockdown in HCC cells was verified by flow cytometry. **C** The arrest of cell cycle in G2 phase in shPOLQ cells was shown by flow cytometry. Data = mean ± SD (n ≥ 3). *** P < 0.001

The results of Celigo cell counting assay showed that, after the infection of shPOLQ, compared with shCtrl group, both SK-HEP-1 and BEL-7404 cells exhibited slower proliferation rate (P < 0.001) compared with shCtrl group (Fig. 3A). The outcomes of flow cytometry assay demonstrated that, percentage of apoptotic cells is apparently increased in shPOLQ group (P < 0.001) in comparison with shCtrl grou (Fig. 3B). On the other hand, the inhibition of cell proliferation by POLQ knockdown could be attributed to the arrest of cell cycle in G2 phase (Fig. 3C). All these results declared that POLQ may be involved in the cell proliferation and cell apoptosis of HCC cells.

### POLQ knockdown suppressed cell migration of HCC cells in vitro

**Fig. 4:**
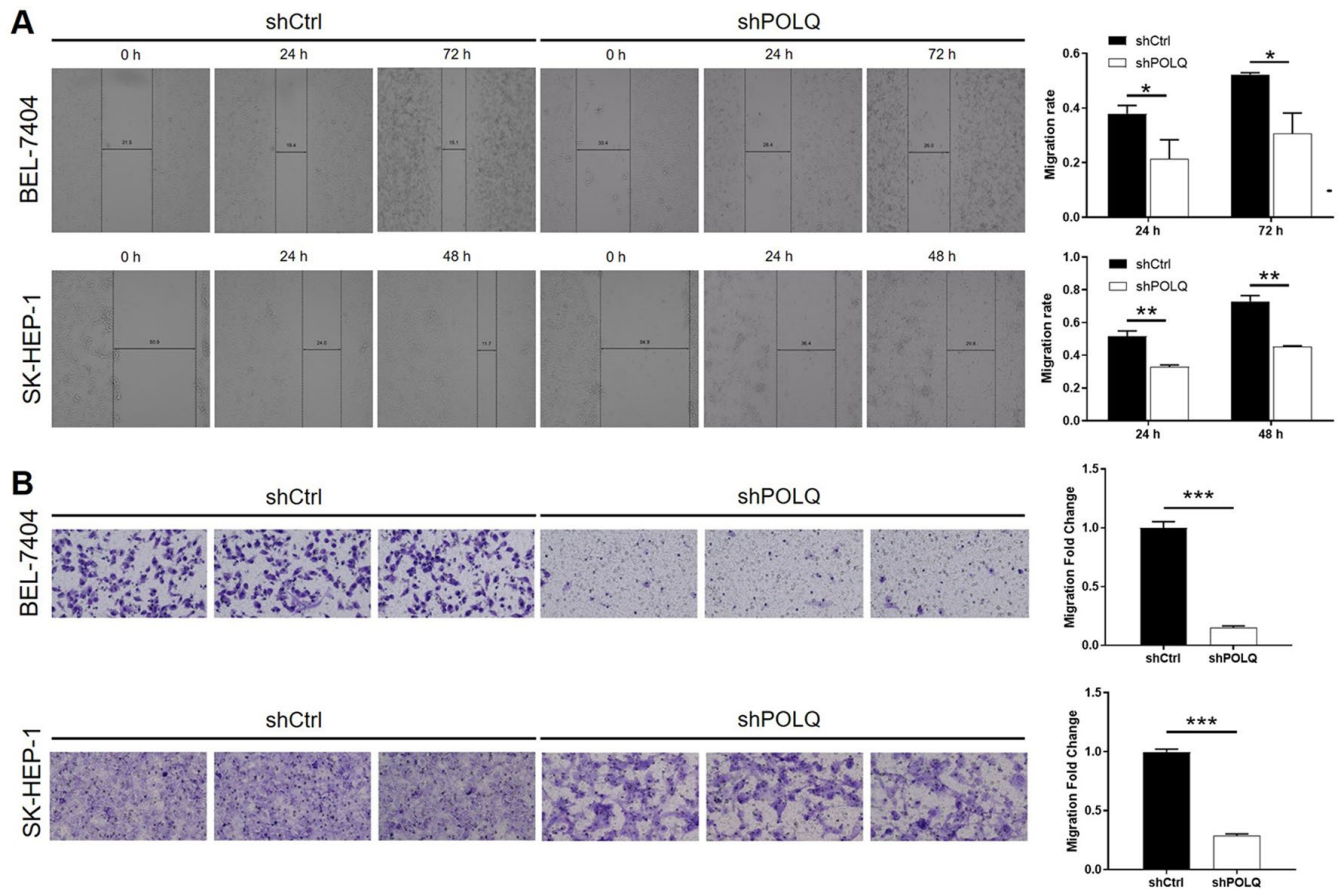
POLQ knockdown suppressed cell migration of HCC cells in vitro. **A** The suppression of cell migration ability by POLQ knockdown in HCC cells was demonstrated by wound-healing assay. **B** The suppression of cell migration ability by POLQ knockdown in HCC cells was demonstrated by Transwell assay. Data = mean ± SD (n ≥ 3). * P < 0.05, ** P < 0.01, *** P < 0.001

The results of wound-healing assay showed that the migration ability of shPOLQ SK-HEP-1 cells was decreased by 38% 48 h after the creation of the wound, and decreased by 41% (P < 0.05) after 72 h of cell culture (Fig. 4A). The results of Transwell assay revealed that, upon the infection of shPOLQ, the migration ability of SK-HEP-1 and BEL-7404 cells could be abated by 71% (P < 0.001) and 85% (P < 0.001), respectively (Fig. 4B). Herein, the results suggested that POLQ may participate in the migration and metastasis of HCC through regulating cell migration.

### Downregulation of POLQ restrained HCC progression in vivo

**Fig. 5:**
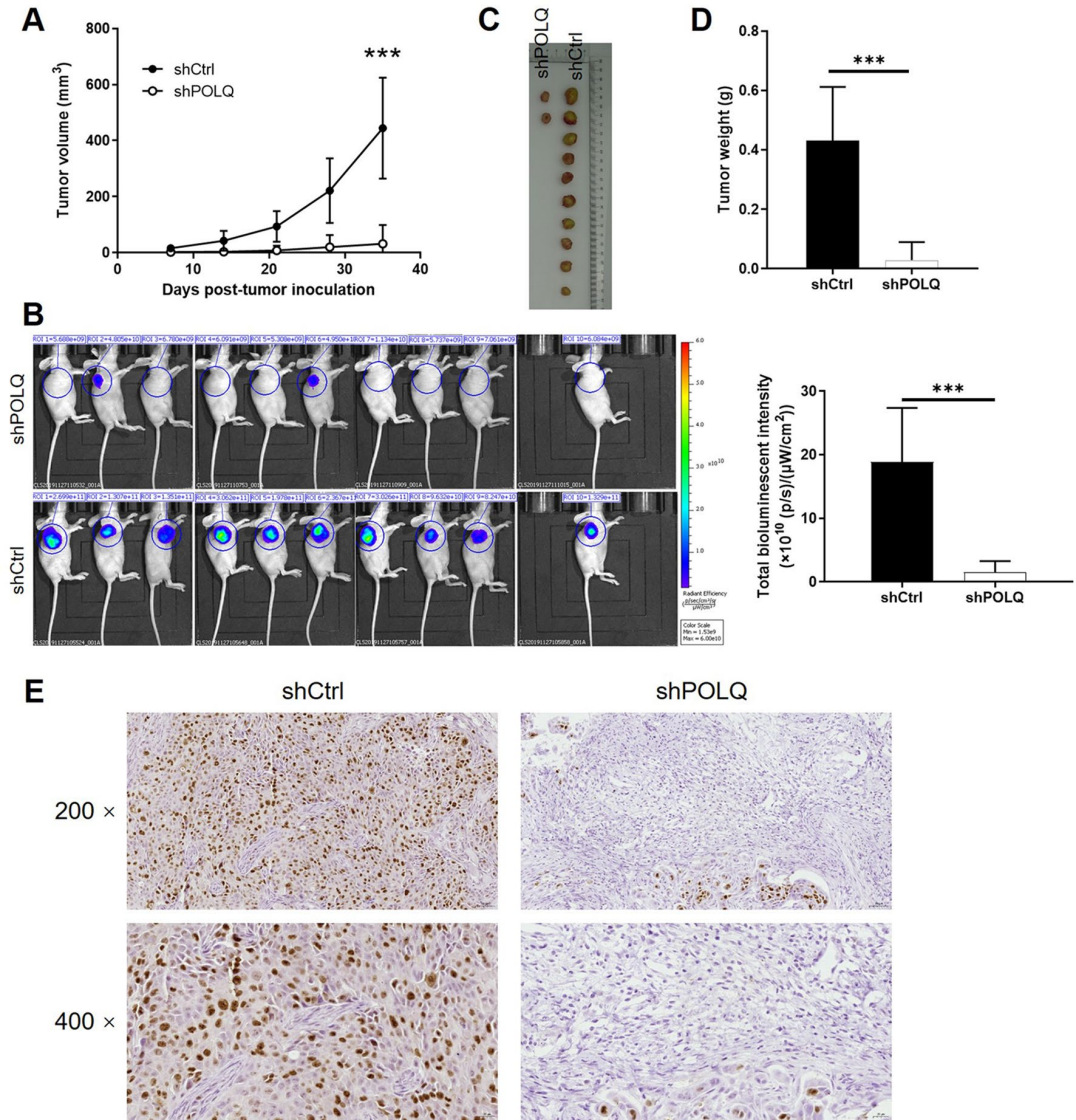
Downregulation of POLQ restrained HCC progression in vivo. **A** Tumor size was measured throughout animal experiments for calculating tumor volume and drawing tumor growth curve, showing the slower growth of tumors in shPOLQ group. **B** In vivo fluorescence imaging was performed before sacrificing animal to observe the tumor burden, which is represented by the fluorescence intensity. **C** Photos of tumors were taken after sacrificing the mice model and removing the xenografts. **D** Weight of xenografts was carried out, recorded and used for comparison between shCtrl and shPOLQ groups. **E** The expression of Ki67 was detected by IHC in xenografts obtained from shCtrl and shPOLQ groups, respectively. Data = mean ± SD (n ≥ 3). *** P < 0.001

SK-HEP-1 cells infected with shPOLQ or shCtrl were injected into nude mouse to further evaluate whether POLQ knockdown can inhibit HCC progression in vivo. The tumor growth curve was drawn based on the measurement of tumor size during animal experiments, showing the relatively slower growth rate of tumors formed by shPOLQ cells (Fig. 5A). Similarly, the in vivo fluorescence of the tumors, derived from GFP on lentivirus vector and detected by in vivo fluorescence imaging, also indicated less tumor burden in shPOLQ group (Fig. 5B). Finally, the tumors were peeled from the mice and subjected to weighing and IHC analysis for detecting Ki67 expression. The results of IHC exhibited that Ki67 expression was greatly repressed by shPOLQ infection (Fig. 5C–E). These data suggested that downregulation of POLQ could inhibit HCC development in vivo.

### Exploration of regulatory mechanism of POLQ on HCC

**Fig. 6:**
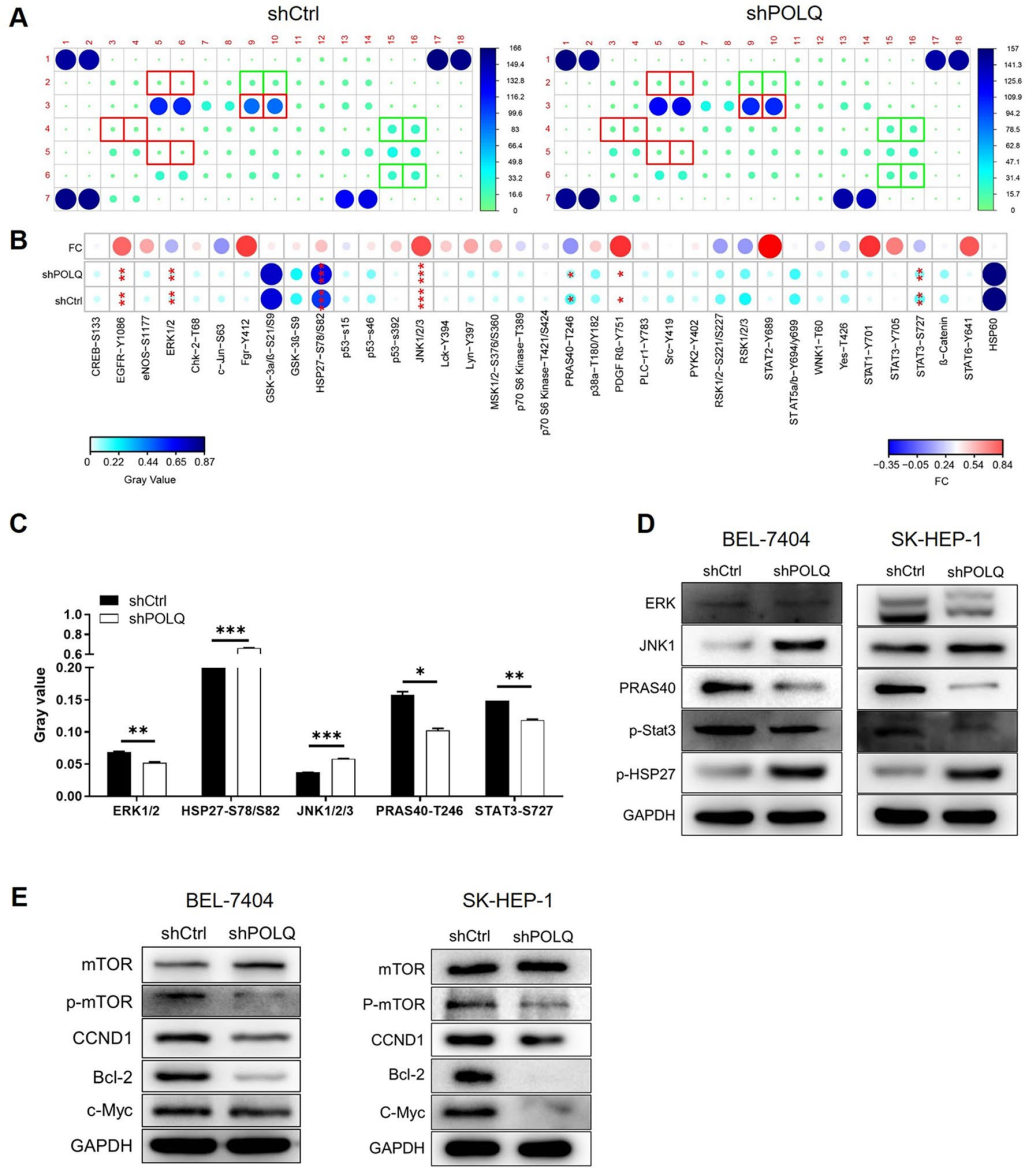
Exploration of regulatory mechanism of POLQ on HCC. **A**, **B** A human apoptosis signaling array was used to detect the difference in the phosphorylation level of various apoptosis related signaling pathways. **C** The proteins with significantly differential phosphorylation level were shown for clearer demonstration. **D** The expression of ERK, JNK1, PRAS40, p-Stat3 and p-HSP27 was detected in both cell lines with or without POLQ knockdown. **E** Expression levels of several tumor-related factors were detected by western blotting. Data = mean ± SD (n ≥ 3). * P < 0.05, ** P < 0.01, *** P < 0.001

In SK-HEP-1 cells with or without POLQ expression interference, expression of proteins in human apoptosis related signaling pathways was detected by an antibody array. It was demonstrated that the phosphorylation of HSP27 at S78/S82 site and JNK1/2/3 was enhanced and that of mTOR, ERK1/2, PRAS40 at T246 site and STAT3 at S727 site were decreased in shPOLQ cells (Fig. 6A–C), indicating the potential involvement of these pathways in the regulation of HCC by POLQ. The regulation of these pathway by POLQ knockdown was also verified by western blot in both BEL-7404 and SK-HEP-1 cell lines (Fig. 6D). Moreover, further investigation revealed the alleviated phosphorylation of mTOR and the downregulation of CCND1, Bcl-2 and c-Myc (Fig. 6E).

## Discussion

In the present study, we found the overexpression of POLQ in HCC, which was associated with higher malignancy and poor prognosis. As one of the members in human DNA polymerase A family, early functional studies on POLQ indicated that its normal expression plays an important role in maintaining gene integrity, preventing mutations caused by defects in base excision repair and inter-strand cross-link. Wang et al*.* reported that POLQ plays an important role in the repair of DNA double-strand breaks caused by fork collapse [13]. Also, POLQ was identified as a key regulator in chromosomal break repair and replication stress response with the help of RAD52 [14]. In the past decades, accumulating evidence showed that POLQ also participate in the development and progression of human cancers because of its critical functions in the repair of genomic double-strand breaks [11, 15]. Previous study showed that POLQ was associated with a concomitant overexpression of firing genes that were significantly related to poor prognosis of colorectal cancer patients [16, 17]. Moreover, POLQ overexpression was found to be associated with advanced pathologic stage, increased somatic mutation load, and PLK4 overexpression in lung adenocarcinoma, thus inducing centrosome amplification [18], indicating the potential involvement of POLQ in the development of lung cancer. Yoon et al*.* found that Error-Prone translesion synthesis dominated by POLQ possesses protective effects against skin cancers [19]. Research by Fernandez-Orgiler et al*.* found that POLQ is a translesion synthesis polymerase involved in parasite DNA damage tolerance, which can confer resistance to macrophage invasion [20]. Feng et al*.* further demonstrate that genetic alterations in PolqSL genes could be observed in around 30% of TCGA breast cancer samples, indicating that inhibiting POLQ could be a promising therapeutic strategy for breast cancer treatment [21]. Despite of these, the role of POLQ in HCC is still not unclear and has not been explored.

In this study, SK-HEP-1 and BEL-7404 cell models with silenced POLQ were established and examined for malignant phenotype. After the infection of lentivirus plasmids prepared for silencing POLQ, the proliferation and migration of HCC cells were significantly inhibited and the cell apoptosis was dramatically increased. Moreover, the percentage of cells in G2 phase increased in shPOLQ group compared with shCtrl group, by which may POLQ knockdown interfered the cell growth. Enhanced cell apoptosis induced by G2/S cell cycle arrest was reported in many kinds of cancers [22]. Furthermore, our work also proved the inhibition of tumorigenesis and tumor growth in vivo upon POLQ downregulation.

So far as we known, there are many signaling pathways associated with liver cancer invasion and metastasis, for instance the phosphoinositide 3-kinase (PI3K)/AKT/mammalian target of rapamycin (mTOR) and rat sarcoma (Ras)/Raf/MEK/extracellular signal-regulated kinase (ERK) signaling pathways, which alters the biological activities of cancer cells through induction of malignant transformation, inhibition of apoptosis, enhancement of proliferative and metastatic capabilities, and eventually leads to the malignant progression of tumors and poor prognosis of the patients [23]. To clarify the regulatory mechanism of POLQ in HCC, the involvement of a series of apoptosis-related signaling pathways was detected in HCC cells with or without POLQ knockdown. The phosphorylation of HSP27 at S78 or S82 site was found to be elevated in shPOLQ cells, which plays an important in suppressing cell apoptosis [24]. Moreover, the phosphorylation, as well as activation of ERK1/2 [25], PRAS40 [26], STAT3 [27] and mTOR [28], which are well-documented signaling pathways playing important role in apoptosis resistance and tumor progression, was significantly alleviated in shPOLQ cells. Moreover, the downregulation of CCND1 [29], Bcl-2 [30] and c-Myc [31] also provide mechanistic evidence for the regulation of HCC development by POLQ.

In conclusion, our results revealed a positive relationship between overexpression of POLQ and HCC development and prognosis. Depletion of POLQ is capable of inhibiting HCC in vitro or in vivo through regulating cell proliferation, cell apoptosis and cell migration. Therefore, POLQ possesses the potential to become a therapeutic target and prognostic indicator of HCC.

## Data Availability

Not applicable.
